# The Genetic Background of Mice Influences the Effects of Cigarette Smoke on Onset and Severity of Experimental Autoimmune Encephalomyelitis

**DOI:** 10.3390/ijms20061433

**Published:** 2019-03-21

**Authors:** Gaby Enzmann, Roberto Adelfio, Aurélie Godel, Neda Haghayegh Jahromi, Silvia Tietz, Sabrina S. Burgener, Urban Deutsch, Hartmut Wekerle, Charaf Benarafa, Britta Engelhardt

**Affiliations:** 1 Theodor Kocher Institute, University of Bern, Freiestrasse 1, 3012 Bern, Switzerland; gaby.enzmann@tki.unibe.ch (G.E.); roberto.adelfio@roche.com (R.A.); aurelie.godel@unibas.ch (A.G.); neda.haghayegh@tki.unibe.ch (N.H.J.); silvia.tietz@tki.unibe.ch (S.T.); urban.deutsch@tki.unibe.ch (U.D.); 2 Institute of Virology and Immunology, Sensemattstrasse 293, 3147 Mittelhäusern, Switzerland; sabrina.burgener@vetsuisse.unibe.ch; 3 Department of Infectious Diseases and Pathobiology, Vetsuisse Faculty, University of Bern, 3012 Bern, Switzerland; 4 Max-Planck-Institute for Neurobiology, Am Klopferspitz 18, 82152 Martinsried, Germany; hWekerle@neuro.mpg.de

**Keywords:** tobacco smoke, risk factor, multiple sclerosis, spontaneous experimental autoimmune encephalomyelitis, and induced experimental autoimmune encephalomyelitis

## Abstract

Multiple sclerosis (MS) is the most common inflammatory disorder of the central nervous system (CNS) in young adults leading to severe disability. Besides genetic traits, environmental factors contribute to MS pathogenesis. Cigarette smoking increases the risk of MS in an HLA-dependent fashion, but the underlying mechanisms remain unknown. Here, we explored the effect of cigarette smoke exposure on spontaneous and induced models of experimental autoimmune encephalomyelitis (EAE) by evaluating clinical disease and, when relevant, blood leukocytes and histopathology. In the relapsing-remitting (RR) transgenic model in SJL/J mice, we observed very low incidence in both smoke-exposed and control groups. In the optico-spinal encephalomyelitis (OSE) double transgenic model in C57BL/6 mice, the early onset of EAE prevented a meaningful evaluation of the effects of cigarette smoke. In EAE models induced by immunization, daily exposure to cigarette smoke caused a delayed onset of EAE followed by a protracted disease course in SJL/J mice. In contrast, cigarette smoke exposure ameliorated the EAE clinical score in C57BL/6J mice. Our exploratory studies therefore show that genetic background influences the effects of cigarette smoke on autoimmune neuroinflammation. Importantly, our findings expose the challenge of identifying an animal model for studying the influence of cigarette smoke in MS.

## 1. Introduction

Multiple sclerosis (MS) is a complex multifactorial disease caused by the interplay of genetic and environmental factors, which drive the inflammatory process in the central nervous system (CNS) causing tissue injury and, if untreated, irreversible disability in MS patients.

Genome-wide association studies (GWAS) using single nucleotide polymorphisms (SNPs) from the HapMap project have allowed for the use of an unbiased approach to search the whole genome and to identify SNPs associated with MS. To this end the International Multiple Sclerosis Genetics Consortium has associated over 230 genetic variants with an increased risk of developing MS [1,2]. Most of the MS-relevant polymorphisms are located either in or close to protein-coding regions directly linked to immune-related functions, supporting the argument that MS is a prototypic T-cell mediated autoimmune disease.

In addition, MS has environmental underpinnings. A large number of recent studies and metadata analysis indicate a positive and consistent correlation between smoking and the development of MS [3,4,5]. The confidence in this correlation is increased by the fact that the risk of MS augments with the cumulative dose of smoking [6,7]. Furthermore, no study has reported an inverse relationship and only very few reported no effect of smoking on risk of MS [8]. In addition, there is evidence that genetic factors and smoking have compounding effects. Studies of HLA genotypes have shown positive correlations between carriage of HLA-DRB1*15 and non-carriage of HLA-A*02 haplotypes with higher risks of developing MS [9]. Of note, the association observed between tobacco smoking and risk of MS were not found in case-control and cohort studies in Northern Europe evaluating the effect of moist snuff [7,10]. This strongly supports that the effect of tobacco is related to the effects of smoke on the lungs rather than to the effects of nicotine only.

Global consumption of cigarettes has increased more than three-fold from 1950 to 2000 [11], a time period during which MS incidence has been observed to significantly rise, particularly in females [12]. According to the World Health Organization, 28% of Europeans are actively smoking and a similar proportion of adults are ex-smokers. Therefore, the individual, societal, and economic burden of tobacco consumption are likely to remain high for the 21st century.

The molecular mechanisms responsible for the association between smoking and MS are not yet known. Smoking has, however, been shown to produce a pro-inflammatory environment in the lung. Burning tobacco yields the release of gaseous and particulate constituents interacting with the immune system of the upper and lower respiratory tract (for review [13]). Specifically, pulmonary epithelial cells respond to cigarette smoke condensate with the production of inflammatory molecules such as the soluble intercellular adhesion molecule (sICAM)-1, granulocyte monocyte colony stimulating factor (GM-CSF), interleukin (IL)-1 and IL-8, and the recruitment of neutrophils and macrophages [14,15,16]. Neutrophils have been underestimated in the pathogenesis of MS and its animal model experimental autoimmune encephalomyelitis (EAE), but recent findings ascribe neutrophils a role as immune mediators and inducers of the maturation of monocyte-derived dendritic cells. The latter cause induction of T-cell proliferation and polarization towards the Th1 phenotype in vitro [17,18,19]. In spontaneous EAE models, increased numbers of circulating neutrophils have been observed in the early phase of the disease [20]. Increased levels of CXCL5, a neutrophil activating chemokine, are detectable in the plasma of relapsing MS patients [20] suggesting a role of neutrophils in EAE and MS pathology. It remains to be investigated if neutrophil infiltration into the lung induced by cigarette smoke contributes to the detrimental role of smoking in MS.

Although smoking is a risk factor that is preventable for MS patients, we consider the identification of the molecular mechanisms on how cigarette smoke contributes to MS pathogenesis significant, as it will provide further insight into the etiology of the disease and thus ultimately offer novel therapeutic targets. Therefore, we explored the effects of cigarette smoke exposure on the disease course of a combination of spontaneous and induced EAE models in C57BL/6J and SJL/J backgrounds to validate their suitability for evaluating the compounding effects of cigarette smoke on MS. We used two spontaneous EAE models established in distinct mouse genetic backgrounds: the relapsing-remitting (RR) SJL mouse EAE model, where SJL/J mice carry a transgenic T-cell receptor (TCR) recognizing MOG_aa92–106_ in the context of MHC class II, I-A^s^ [21] and the optico-spinal encephalomyelitis (OSE) C57BL/6J mouse model, where C57BL/6J mice carry both a transgenic T-cell receptor (TCR) recognizing MOG_aa35–55_ in the context of MHC class II I-A^b^ and the gene coding for the rearranged IgH variable chain of the classical anti-MOG monoclonal antibody, 8.18-C5 (IgH^MOG^ mice) [22]. We also employed actively induced EAE models in SJL/J mice that closely resemble the relapsing-remitting disease course of MS and in C57BL/6J mice, which exhibit a chronic disease progression.

## 2. Results

### 2.1. Disease Incidence of Spontaneous EAE in the RR Mouse Model is too Low to Study Effects of Cigarette Smoke on Disease Development

**Figure 1:**
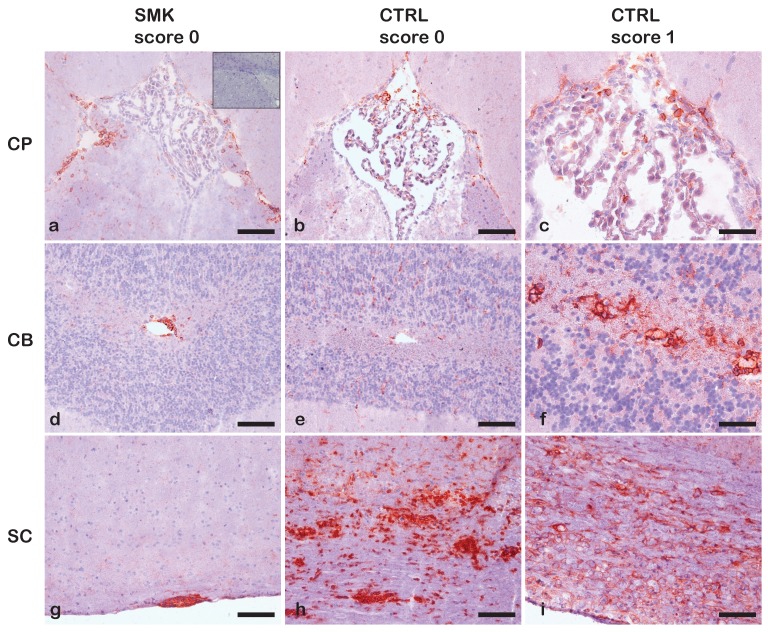
Distribution of CD45^+^ inflammatory cells in the CNS of the RR mice exposed to cigarette smoke or air for 18 weeks. Immunostaining for CD45^+^ inflammatory cells in areas of the choroid plexus (CP) of the 3rd ventricle (**a**–**c**), in the cerebellum (CB) (**d**–**f**) and spinal cord (SC) (**g**–**i**) of RR mice exposed to cigarette smoke (SMK) or air (control = CTRL) for 18 weeks is shown for comparison. Scarce CD45^+^ inflammatory infiltrates in the vicinity of the choroid plexus (CP) of the 3rd ventricle and in the cerebellum (CB) are visible in the SMK and CTRL mouse with clinical score 0 (**a**,**b**,**d**,**e**). These CD45^+^ infiltrates are more prominent in the CTRL mouse with a clinical score 1 (**c**,**f**). CD45^+^ cellular infiltrates in the spinal cord of SMK RR mice were rather small and preferentially detected in the thoracic spinal cord white matter (**g**). In the same spinal cord location, CD45^+^ cellular infiltrates were prominent in the CTRL group irrespective of the clinical score (**h**,**i**). Sections were counterstained with hematoxylin. A negative staining control employing an isotype-matched antibody is shown as inset in panel (**a**). Low disease incidence in RR mice prohibited quantitative analysis of the CNS infiltrates. There were two mice in the CTRL and a single mouse in the SMK group that displayed clinical signs of EAE at the end of the experiment. The scale bars in panels (**a**,**b**), (**d**,**e**) and (**g**,**h**) are 100 μm and in panels (**c**,**f**,**i**) are 50 µm.

We first investigated the effects of cigarette smoke exposure on the development of spontaneous relapsing remitting EAE in two independent experiments in the RR mouse model with the SJL/J background. Ten female RR SJL/J mice were randomly assigned to groups exposed to cigarette smoke (SMK) or room air (CTRL) from six weeks of age. Mice were monitored for disease incidence for 18 weeks. In the first experiment, only one out of five mice in the SMK group and two out of five mice in the CTRL group developed clinical signs of EAE. In a second identically designed experiment with four mice per group, none of the mice developed spontaneous EAE during the observation period of 18 weeks. Overall, this equals a disease incidence of 11% in the SMK group and 22% in the CTRL group, suggesting that cigarette smoke exposure does not worsen EAE incidence or severity in this model. Histological analysis of brain and spinal cord tissue of RR mice with a clinical score of “0” revealed very few CD45 positive inflammatory cells in the choroid plexus of the 3rd ventricle and in the cerebellum of either experimental group at the end of the experiment (Figure 1a,b,d,e). On the contrary, inflammation in the spinal cord white matter was only evident in the CTRL group (Figure 1h). An increase in the EAE clinical score positively correlated with the extent of inflammation in both the brain and spinal cord in a mouse of the CTRL group (Figure 1c,f,i).

Taken together, our observations suggest that the RR mouse model is not well suited to studying the effect of smoke exposure on EAE pathogenesis in a way that it would reflect increased MS incidence caused by cigarette smoke exposure.

### 2.2. Early Onset of Spontaneous EAE in the OSE Model in the C57BL/6J Background Prevents Studying the Effect of Cigarette Smoke

**Figure 2:**
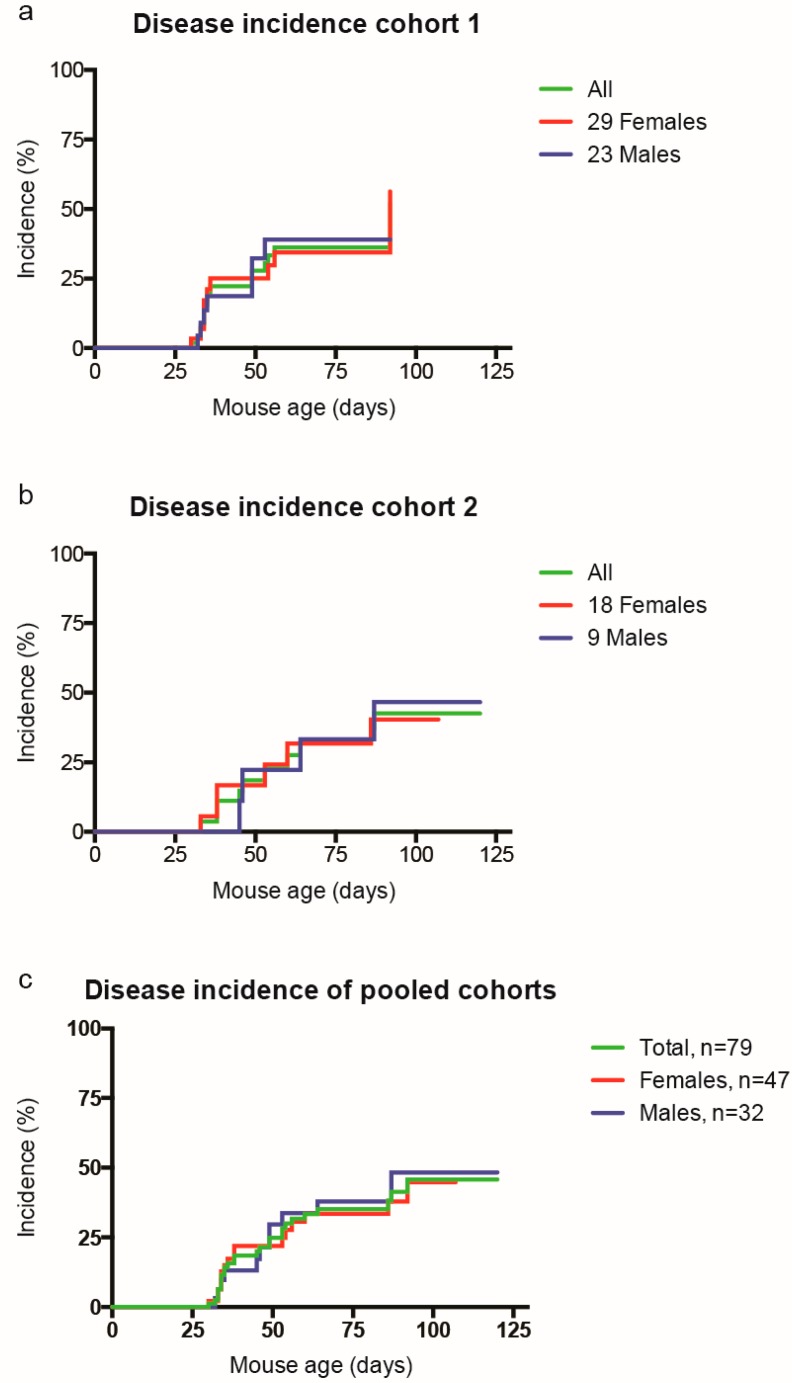
Early disease onset in the OSE mouse line precludes smoke exposure experiments. Onset of disease was monitored on two cohorts of OSE mice housed in separate breeding rooms. (**a**) Disease onset of cohort 1 monitored for up to 90 days postpartum (*n* = 52; 29 females and 23 males). (**b**) Disease onset for cohort 2 monitored for up to 120 days (*n* = 27; 18 females, 9 males). (**c**) Pooled data from the two cohorts. Disease incidence curves from the pooled data of the two cohorts were compared for males and females and showed no statistical difference (Logrank test) between sexes.

In the process of breeding sufficient OSE mice to investigate the influence of cigarette smoke on the development of spontaneous EAE in the C57BL/6J background, we quickly realized that disease onset in this spontaneous EAE model conflicted with our established smoke exposure regimen starting at the age of six weeks. Twenty percent of the female OSE C57BL/6J mice already displayed clinical signs of EAE at that time point. This prompted us to assess disease onset in individual OSE mice of both sexes raised in two different breeding units of the local animal facility. One cohort was monitored for up to 90 days postpartum (*n* = 52; 29 females and 23 males) (Figure 2a) and a second cohort for up to 120 days (*n* = 27; 18 females, 9 males) (Figure 2b). Combined evaluation of a total of 79 OSE mice exposed to room air (47 females, 32 males) confirmed an early disease onset (Figure 2c), which precluded conducting significant smoke exposure experiments with this mouse line.

### 2.3. Cigarette Smoke Exposure Induces Protracted Disease with a Delayed Onset in PLP_aa139–153_ - Induced EAE in SJL Mice

**Figure 3:**
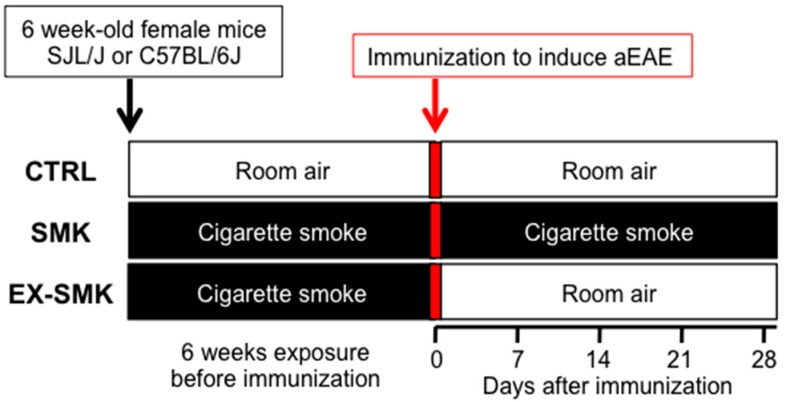
Experimental setup. Sequence of smoke exposure experiments in actively induced EAE mouse models is shown.

**Figure 4:**
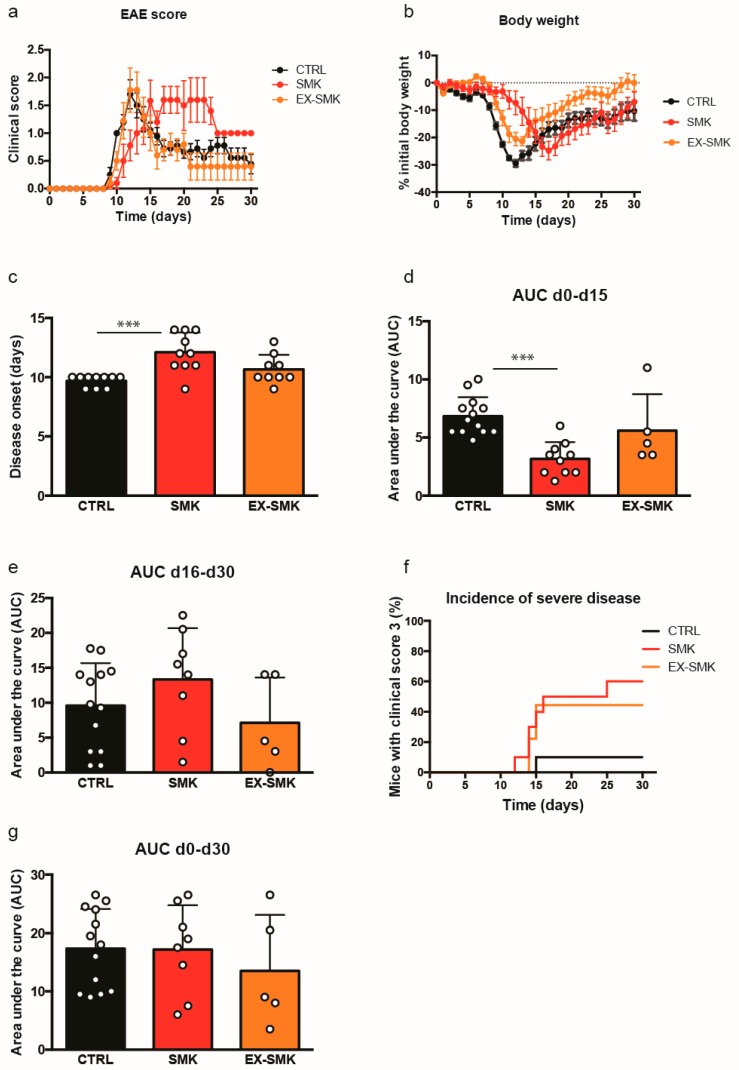
Cigarette smoke exposure delays active EAE onset and remission in SJL mice. Groups of SJL/J females were exposed to room air or cigarette smoke as shown in Figure 3. Clinical score (**a**), body weight (**b**) and disease onset (**c**) are shown for SJL/J mice immunized with PLP (*n* = 9–10 mice/group). Overall disease severity of mice from experiment 1 and experiment 2 was compared by calculating the area under the curve (AUC) between day 0–15 pI (**d**) (*p* < 0.05, unpaired t-test), 16–30 pI (**e**), and for the whole duration of the experiment day 0–30 (**g**) (*n* = 23 mice/group). (**f**) Incidence of severe disease is shown as the fraction of mice with a clinical sore of 3 (experiment 1: *n* = 9–10/group). Data are shown as mean ± SEM (**a**–**g**) and statistical differences between the groups for disease onset and AUCs (**c**–**e**,**g**) were analyzed using the t-test (* *p* < 0.05).

Next, we studied the effect of cigarette smoke on actively induced EAE. We used an active relapse-remitting EAE model induced in SJL/J mice by immunization with PLP_aa139–153_ in CFA. Mice were randomized in three groups (*n* = 10), where they were exposed to room air (CTRL group) or to cigarette smoke for 6 weeks before immunization (SMK and EX-SMK). After immunization, mice in the SMK group were subjected to further smoke exposure, while the CTRL and EX-SMK were exposed to room air (Figure 3). Further smoke exposure after immunization (SMK group) led to a significantly delayed disease onset compared to the CTRL (Figure 4a–c). The peak of clinical EAE appeared delayed but prolonged in the SMK group compared to CTRL as shown by the absence of an early peak in disease score, extended duration of clinical EAE above score 1 (Figure 4a), and a delayed nadir for body weight loss (Figure 4b) in the SMK group. The EX-SMK group showed an intermediate phenotype between the CTRL and SMK group for disease severity; onset was not significantly different from CTRL (Figure 4a–c).

We repeated the study including only CTRL and SMK groups (*n* = 20–21). Animals were pre-assigned to subgroups to be euthanized at days 7, 14, 21, 28, and 30 days for leukocyte and histological analysis. The delayed disease onset and reduced initial severity of EAE in the SMK group was replicated in the second experiment. Combining mice from the first experiment and mice pre-assigned to be euthanized at day 30 from the second experiment, we quantified overall disease severity by calculating the area under the curve (AUC). Disease was significantly more severe in CTRL than SMK mice in the early phase (days 0–15 pI) of the disease (Figure 4d). In the late phase (days 16–30 pI), we observed more severe disease in the SMK vs CTRL, however, this difference did not reach statistical significance (Figure 4e). Increased disease severity in the SMK compared to WT group as observed in the first experiment is better shown by the number of animals reaching a clinical score 3 (Figure 4f). Because animals reaching a score of 3 were euthanized according to our animal experimentation permit, the most severe animals in this group were not represented in the evaluation of the AUC. Thus, overall disease severity over the whole period was not different between the groups.

**Figure 5:**
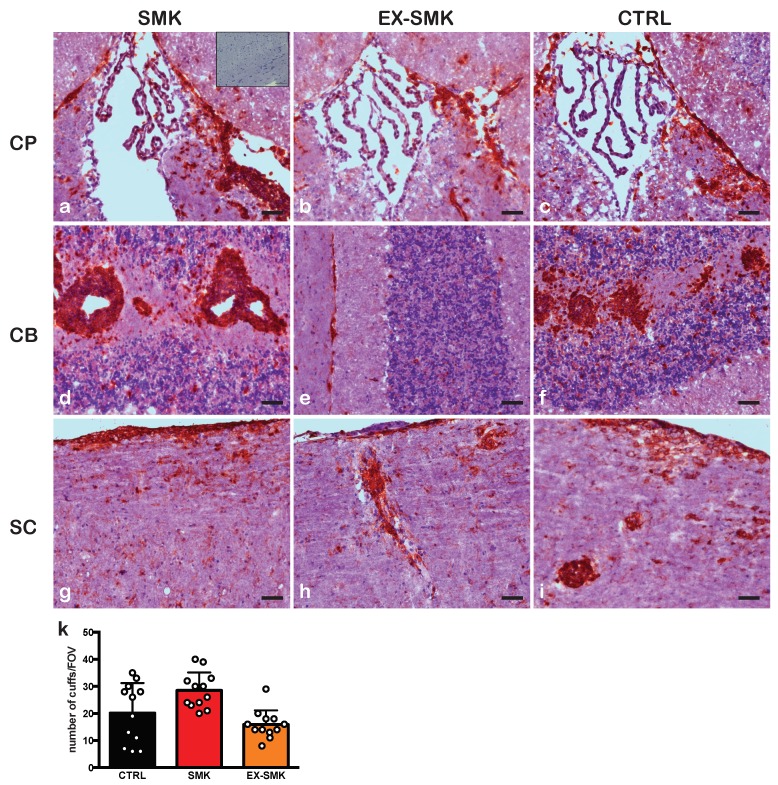
Comparable CNS localization and accumulation of CD45^+^ inflammatory cells between SMK, EX-SMK, and CTRL groups in SJL mice subjected to aEAE. Immunohistochemical staining for CD45^+^ leukocytes in the vicinity of the choroid plexus (CP) of the 3rd ventricle (**a**–**c**), the cerebellum (CB) (**d**–**f**), and the spinal cord (SC) (**g**–**i**) of SJL/J mice (day 31 post immunization, clinical score 1) is shown. Quantitative analysis of the cerebellar infiltrate (**k**) demonstrated more and bigger inflammatory cuffs in the cerebellum of the SMK versus the EX-SMK and CTRL group. Differences were not significant. Sections were counterstained with hematoxylin. A negative staining control employing an isotype-matched antibody is shown as inset in panel a. The scale bars are 100 µm.

Immunohistochemical analysis of brain and spinal cord specimens at 30 days after EAE induction revealed the presence of massive CD45 positive immune cell infiltrates adjacent to the choroid plexus of the 3rd ventricle (Figure 5a–c), in the parenchyma and the meninges of the cerebellum (Figure 5d,f), and in the spinal cord (Figure 5g–i) of all experimental groups without visible differences. A notable difference was observed in the cerebellum of the EX-SMK group (Figure 5e). Quantitative analysis of CNS infiltrate revealed a trend towards higher numbers of inflammatory cuffs in the cerebellum of the SMK versus the CTRL and EX-SMK groups. Differences were not statistically significant (Figure 5k).

**Figure 6:**
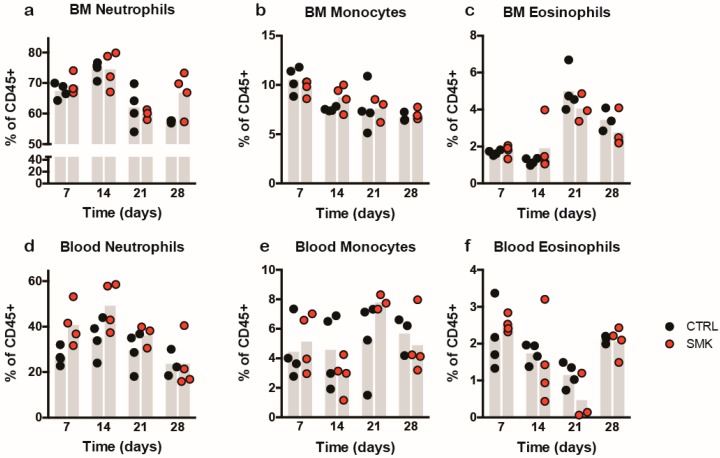
Leukocyte analysis in bone marrow and blood of SJL/J mice revealed a small but not significant increase in blood neutrophils of SMK mice at days 7 and 14 after immunization. Percentage of leukocyte subsets in bone marrow (BM) (**a**–**c**) and blood (**d**–**f**) were analyzed using 4-color flow cytometry gating of total leukocytes (CD45^+^). The subsets were identified as neutrophils (CD11b^+^Ly6G^+^), monocytes (CD115^+^), and B cells (CD19^+^). A transient, but not statistically significant, increase in the percentage of peripheral neutrophils in SMK mice compared to CTRL is noted at days 7 and 14 post immunization (**d**).

To determine, if cigarette smoke exposure influences the number of circulating innate immune cells, we analyzed bone marrow and peripheral blood leukocytes in a subset of predefined animals at 7, 14, 21, and 28 days post EAE induction (Figure 6). Cigarette smoke did not affect the output of neutrophils, monocytes, and eosinophilic granulocytes from the bone marrow (BM) post EAE induction in SJL/J mice (Figure 6a–c). Neutrophils and monocytes were described to accumulate in the periphery in response to granulocyte colony-stimulating factor (G-CSF) and the chemokine CXCL1 prior to onset of EAE and their subsequent infiltration into the CNS [20]. The percentage of neutrophils amongst circulating CD45^+^ blood cells was transiently, albeit not significantly, increased at 7 and 14 days pI in the SMK versus the CTRL group, while more similar levels in both groups were observed again at days 21 and 28 pI (Figure 6d). The cigarette smoke induced increase of circulating neutrophils was thus observed during the time when clinical EAE was ameliorated compared to the CTRL group. Therefore, it is difficult to correlate the observation with cigarette smoke induced effects on EAE development in aEAE in the SJL/J mouse.

### 2.4. Cigarette Smoke Exposure Ameliorates MOG_aa35–55_ Induced EAE in C57BL/6J Mice

**Figure 7:**
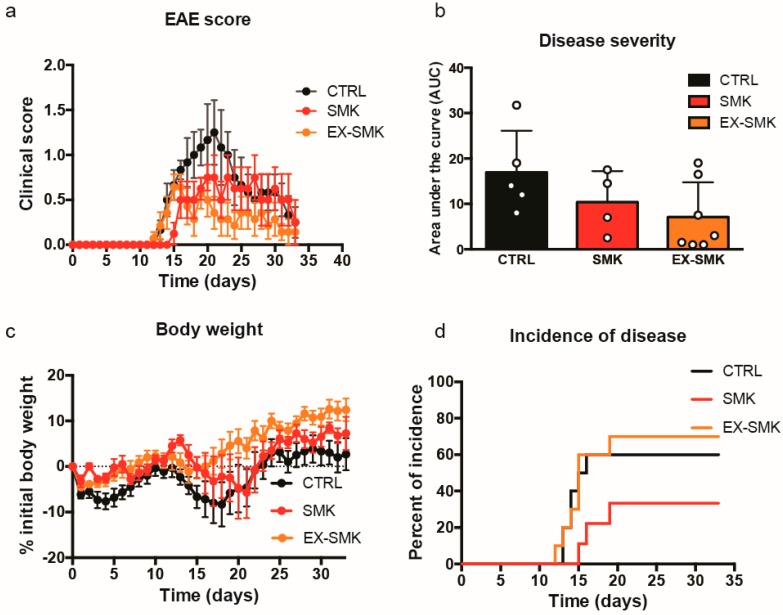
Cigarette smoke exposure ameliorates clinical EAE in C57BL/6J mice. Groups of C57BL/6J females were exposed to room air or cigarette smoke as shown in Figure 3. Clinical score (**a**), disease severity (**b**), body weight (**c**), and disease onset (**d**) are shown following immunization with MOG_aa35–55_ (*n* = 9–10 mice/group). Disease severity based on clinical scores was compared by calculating the area under the curve (AUC). Data are shown as mean ± SD (**a**–**c**) including individual values in (**b**). Individual values “score 0” are not shown for better visibility. Differences between the groups for disease severity (AUC) showed no statistical differences using one-way ANOVA. A Kaplan‒Meier curve for disease incidence showed a significant reduction for the SMK group compared to CTRL using log rank test (*p* < 0.05).

Finally, we investigated the effect of cigarette smoke exposure in a chronic EAE model induced by immunization with myelin oligodendrocyte glycoprotein (MOG) _aa35–55_ in CFA in C57BL/6J mice. Reminiscent of the findings in actively induced EAE in SJL/J mice, cigarette smoke exposure delayed the disease onset compared to the respective control group (Figure 7a). In contrast to actively induced relapsing-remitting EAE in the SJL/J mouse, the chronic aEAE disease course in C57BL/6J mice remained ameliorated in mice exposed to cigarette smoke (Figure 7b). Cessation of smoke exposure at the time of immunization further suppressed the disease progression and was accompanied by the lowest weight loss observed in all groups (Figure 7c). Of note, disease incidence was statistically reduced in the SMK group compared to the CTRL group (Figure 7d), indicating that fewer mice (25%) developed any disease symptoms compared to CTRL (>50% incidence). Immunohistochemical analysis of CNS infiltrates at the end of the experiment revealed only little inflammation in the brain of mice from either experimental group. CD45^+^ cellular infiltrates localized primarily to the meninges and the white matter of the spinal cord indistinguishable in all groups (data not shown). Our findings point to a protective component in cigarette smoke not exclusively acting on infiltration of inflammatory cells into the CNS and suggest interference of smoke exposure with the immunization process.

## 3. Discussion

### 3.1. Spontaneous EAE Models

The advantage of employing spontaneous EAE animal models constitutes the relinquishment of the use of artificial compounds to induce this specific disease.

In a first line of experiments, we thus aimed to investigate the influence of cigarette smoke exposure on onset, disease progression, and pathological features of EAE employing the spontaneous relapse-remitting SJL (RR) and the optico-spinal C57BL/6 (OSE) experimental autoimmune encephalomyelitis models [21,22]. In the study of Pollinger and colleagues [21], the disease incidence for female RR mice was reported to be 80% within 35–160 days after birth. However, this yield was not achieved in the population of female RR mice bred under SPF conditions at our animal facility. Here, only 22% (2/9 mice) of the females in the air CTRL group exhibited clinical signs of EAE by the end of the experiment at 24 weeks of age. This equals 20% of the theoretical expected yield. More importantly, we found that only 11% of RR females exposed to cigarette smoke developed clinical disease. Thus, we can conclude that smoke exposure did not increase disease incidence in the RR model. Furthermore, the overall low disease incidence precluded any meaningful exploration of changes induced by cigarette smoke on pathological mechanisms of this model. Thus, investigating potential mechanisms of cigarette smoke induced alterations of EAE in the RR model would require large cohorts of mice, which is in disagreement with the 3R rules aiming at reducing animal experimentation. We therefore refrained from extending studies in this model.

With the OSE model, the investigation of the effect of cigarette smoke exposure on the development of EAE was prevented by the early onset of the disease. Our established smoke exposure regimen [23] foresaw smoke exposure at an age of six weeks for a total of 18 weeks. Unfortunately, 20% of all OSE mice bred at the local animal facility already displayed clinical signs of EAE at this time point. This did not contradict earlier reports by Krishnamoorthy and colleagues indicating a mean disease onset at 6 ± 2 weeks with 51% of the mice developing spontaneous OSE EAE within 120 days after birth [22]. We did not observe any difference in the disease onset between the sexes. Starting smoke exposure at an earlier time point or exposing pregnant dams to smoke was rejected as it might interfere with mounting a sufficient immune response due to the relative immaturity of the adaptive immune system at that age (for overview [24]). Altogether, the temporal profile of disease incidence in the OSE mouse line renders it unsuitable for sustained smoke exposure experiments.

### 3.2. Actively Induced EAE Models

In a second approach, we evaluated the influence of smoke exposure on the development of actively induced EAE in two different models. We found that cigarette smoke exposure delayed the onset of aEAE in both the SJL/J and C57BL/6J background. These results may be explained in part by the effect of nicotine. Nicotine, one component of cigarettes, was attributed both pro- and anti-inflammatory effects on the immune system. Its signaling pathway [25,26,27] and cellular response has been investigated in several autoimmune diseases (for review [28]), including EAE [29,30]. The cholinergic system dampens the peripheral immune response indicating the involvement of the α7- nicotinic acetylcholine receptor (nAChR) [25,26], which is found both on neurons and the microglia. In an active EAE model in C57BL/6 mice, Hao and colleagues [27] indicated that additional non-α7 nAChR subtypes are involved in dampening neuroinflammation in the presence of nicotine, which was subsequently confirmed by Simard and colleagues [31]. Prophylactic application of nicotine ameliorated active EAE disease severity in C57BL/6 mice by a reduction of demyelination and counteracting disease accompanying weight loss [29]. Likewise, in our present study we observed that the extent of weight loss associated with the onset of EAE was reduced in mice exposed to cigarette smoke before disease induction (EX-SMK group) and in those continuously exposed to cigarette smoke (SMK group) in both SJL/J and C57BL/6J background. Extended smoke and therefore nicotine exposure after EAE induction continued to show some effect on weight preservation in our SJL/J and C57BL/6J SMK groups but might be outweighed by cigarette smoke condensate (CSC) negatively affecting clinical disease progression [29]. These results allow speculation on a beneficiary role for nicotine in cigarette smoke on EAE disease progression in both SJL/J and C57BL/6J mice as long as smoke exposure ceased at the time of immunization. Continued smoke exposure after immunization led to strain specific effects regarding EAE disease progression. In detail, the disease was protracted and showed a trend to increased severity in SJL/J EAE mice but was ameliorated in the C57BL/6 background. This might point to differences between both mouse strains regarding the nicotine modification of microglia via α7- nAChR towards a neuroprotective phenotype [32]. Nicotine furthermore targets additional myeloid subpopulations as it was shown to inhibit infiltration of proinflammatory monocytes and neutrophils into the CNS via α7 and α9 nAChR subtypes during actively induced EAE in C57BL/6J mice [33]. Thus, it is tempting to speculate that nicotine exerts different effects in C57BL/6J versus SJL/J mice.

## 4. Material and Methods

### 4.1. Experimental Autoimmune Encephalomyelitis

*Spontaneous EAE mouse models:* We employed a chronic relapse-remitting (RR) model in which SJL/J mice carrying a transgenic T-cell antigen receptor (TCR) recognizing MOG_aa92–106_ in the context of MHC class II, I-A^s^ develop spontaneous EAE that is initiated by the transgenic CD4^+^ T cells infiltrating the CNS and by MOG-autoantibody producing B cells activated from the natural immune repertoire [21]. Starting with individual mice from the age of 5 to 6 weeks, EAE incidence was reported to reach >80% in females and >60% in males within 160 days after birth [21]. Throughout the experiment we utilized hemizygous females derived from the mating of heterozygous founders with SJL/J wildtype mice. We investigated a total of 18 mice.

In the optico-spinal encephalomyelitis (OSE) model, C57BL/6J mice carry a transgenic T-cell antigen receptor (TCR) recognizing MOG_aa35–55_ in the context of MHC class II I-A^b^ and the gene coding for the rearranged IgH variable chain of the classical anti-MOG monoclonal antibody, 8.18-C5 (IgH^MOG^ mice) [22]. The OSE mice were obtained by crossing C57BL/6-Igh-J(tm1Aigl) single transgenic mice [34] with single transgenic Tg(Tcra2D2,Tcrb2D2)1Kuch mice [35]. In this mouse model, mean disease onset was observed at 6 ± 2 weeks with 51% of mice developing spontaneous optico-spinal EAE (OSE) within 120 days after birth [22]. Experimental mice were double transgenic, i.e., heterozygous for each allele. We followed the disease progression in a group of 79 mice consisting of 47 females and 32 males.

The spontaneous models innately develop EAE with the OSE line reminiscent of a human variant of multiple sclerosis, neuromyelitis optica. The experimental setup for both spontaneous mouse models implied smoke exposure five times a week for 18 consecutive weeks starting at six weeks of age. The respective control groups were exposed to room air entirely. Mice were scored daily prior to smoke exposure according to the classic EAE disease determination: 0, healthy animal; 0.5, animal with a flaccid tail; 1, animal with impaired righting reflex and/or gait; 2, animal with paraplegia; 3, animal with paraplegia and incontinence [36]. Mice exhibiting a clinical score of “0.5” were considered sick and counted positive for disease onset (Table 1). According to our animal experimentation permit, mice with a disease score above 2 had to be euthanized immediately but were included in the analysis. The spontaneous disease developing in RR-SJL/J mice was described to differ dramatically from classical EAE, especially during onset where mice showed signs of ataxia rather than classical EAE symptoms [21]. Ataxic scoring was adapted as published: score 0, healthy; 1, mouse partly tilted, feet fall into cage fence; 2, mouse tilted and tumbling; 3, mouse heavily tilted and moving in circles. Mice exhibiting a clinical score of “1” were considered sick and counted positive for disease onset (Table 2). Due to ethical concerns, animals displaying a disease score above 2 were euthanized immediately.

The area under the curve (AUC) for the clinical severity of EAE was calculated by doing a definite integral of the clinical score graph between two given timepoints (here, the AUC was determined for each curve between 0–30, 0–15, and 16–30 days). To determine the AUC of a clinical score curve y = f(x) of a given treatment group between x = day a and x = day b was integrated between the limits a and b of the x-axis. This calculation produces a number without a unit. AUC calculation and analysis were performed with GraphPad Prism Software 6. The AUC as a cumulative measure of disease severity in a specific cohort can be compared between different groups [36,37].

*Induced mouse EAE models (active EAE, aEAE)*: Female SJL/J and C57BL/6J wild type mice at six weeks of age were randomly assigned to three experimental groups. The control (CTRL) group respired room air exclusively. In parallel, the other groups were exposed to side and main stream cigarette smoke five days a week for 6 h for a total of six week. Subsequently, active EAE was induced by immunization with proteolipid protein peptide (PLP_aa139–151_) in complete Freund’s adjuvant (CFA) in SJL/J mice as previously described [38,39]. Accordingly, active EAE in C57BL/6 mice was induced by immunization with myelin oligodendrocyte glycoprotein peptide (MOG_aa35–55_) emulsified in complete Freund’s adjuvant and Bordetella pertussis toxin as previously described [38,39]. Post immunization, the smoke exposed (SMK) groups resumed their smoking schedule, whereas the third group was switched back to room air (EX-SMK) along with the controls for the remainder of the experiment. EAE progression was followed for 30 days pI as described above. We investigated a total of 38 C57BL/6J and 60 SJL/J mice, respectively.

### 4.2. Statistics

Differences of the severity of clinical EAE between groups were evaluated using the calculation of the area under the curve (AUC). Onset of disease was calculated for all mice included in an experiment. AUC was analyzed for all mice alive during the entire time period analyzed. Mean values were calculated and significance was evaluated using the t-test (GraphPad Prism6). By calculating the AUC for each mouse, we used all data points accumulated over the respective days of measurements applying the EAE score criteria described in Table 1 and Table 2.

### 4.3. Smoke Exposure

Whole body cigarette smoke exposure was performed using a TE-10z smoking machine (Teague Enterprise) and standard 3R4F ultra-low tar research cigarettes (University of Kentucky, Lexington, KY, USA). The smoking apparatus delivers a mixture of mainstream (11%) and sidestream (89%) cigarette smoke to four inhalation chambers, which can hold up to six standard mouse cages with 5 mice/cage. These individual cages are equipped with a grid providing water and food during exposure. Mice were placed in the smoke exposure chamber six hours/day for five days/week. The smoking cycle constituted of five hours smoke at 100 mg/m^3^ of particulate matter followed by one hour of room air as previously described [23]. The mice were not subjected to physical restraint at any time. Upon finishing the daily smoke exposure routine mice were returned to their home cages and closely monitored for wellbeing. Control mice were not exposed to cigarette smoke and respired room air exclusively.

### 4.4. Tissue Processing and Immunohistology

After completing 18 weeks of smoke exposure or respiration of room air in the spontaneous EAE models and six weeks plus 30 days in the actively induced EAE models, respectively, mice were deeply anesthetized with isoflurane and perfused with 1% paraformaldehyde in PBS, pH 7.4. Brain and spinal cord were dissected and embedded in Tissue-Tek (OCT compound). Immunostaining was performed on 6 µm cryostat sections using a three-step immunoperoxidase staining kit and developed with AEC substrate (Vectastain, Vector) using the following antibodies: rat anti-mouse ICAM-1 (clone 25ZC7) [40], rat anti-mouse ICAM-2 (3C4) (BD Pharmingen, San Jose, CA, USA), rat anti-mouse VCAM-1 (2A11.12) [41], rat anti-mouse PECAM-1 (clone Mec13.3) (BioLegend), rat-anti mouse Ly6G (1A8) [42], rat-anti mouse CD11b/Mac-1 (M1/70) (BD Pharmingen), rat anti-mouse CD4 (GK1.5) (BD Pharmingen), rat anti-mouse CD8 (Lyt2) (BD Pharmingen), rat anti-mouse CD45 (M1/9) (BD Pharmingen), rat CD45R/B220 (RA3-6B2) (BD Pharmingen), rat anti-mouse F4/80 (BD Pharmingen). An isotype-matched rat anti-human CD44 antibody (clone 9B5, BD Pharmingen) was employed as negative staining control. Finally, sections were counterstained with Mayer’s hemalum and assessed using a Nikon Eclipse E600 microscope equipped with a digital camera. Quantification of CNS infiltrate was performed by counting CD45^+^ positive cuffs in the cerebellum of mice of the SMK, EX-SMK, and CTRL group (*n* = 2/group). The number of cuffs was calculated per cerebellar section and is provided as mean ± SD.

### 4.5. Flow Cytometry Analysis

Bone marrow and peripheral blood subsets were isolated 7, 14, 21, and 28 days post immunization and exposure to smoke or room air as published in [43]. The data were analyzed using FlowJo (FlowJo LLC, Ashland, OR, USA).

## 5. Conclusions

Our exploratory study provides evidence that cigarette smoke exposure differentially affects the course of actively induced EAE in different mouse strains underscoring that the genetic background influences the effect of cigarette smoke on autoimmune neuroinflammation. At the same time, the observed ameliorating effects exerted by cigarette smoke on EAE development expose the challenge of identifying an animal model allowing the investigation of the mechanisms by which cigarette smoking impacts on MS pathogenesis.

## Figures and Tables

**Table 1:** Disease score determination for OSE and induced EAE models.

Score	Clinical Criteria
0	No symptom
0.5	Flaccid tail
1	Impaired righting reflex and/or irregular gait
2	Paraplegia
3	Paraplegia and incontinence

**Table 2:** Disease score determination criteria for the SJL/J-RR model.

Score	Clinical Criteria
0	No symptom
1	Partly tilted body, feet fall into cage when walk on grid
2	Tilted body and tumbling
3	Heavily tilted body/head and moving in circles
